# Wireless distributed functional electrical stimulation system

**DOI:** 10.1186/1743-0003-9-54

**Published:** 2012-08-09

**Authors:** Nenad S Jovičić, Lazar V Saranovac, Dejan B Popović

**Affiliations:** 1 School of Electrical Engineering, University of Belgrade, Belgrade, Serbia; 2 Department of Health Science and Technology, Aalborg University, Aalborg, Denmark

## Abstract

**Background:**

The control of movement in humans is hierarchical and distributed and uses feedback. An assistive system could be best integrated into the therapy of a human with a central nervous system lesion if the system is controlled in a similar manner. Here, we present a novel wireless architecture and routing protocol for a distributed functional electrical stimulation system that enables control of movement.

**Methods:**

The new system comprises a set of miniature battery-powered devices with stimulating and sensing functionality mounted on the body of the subject. The devices communicate wirelessly with one coordinator device, which is connected to a host computer. The control algorithm runs on the computer in open- or closed-loop form. A prototype of the system was designed using commercial, off-the-shelf components. The propagation characteristics of electromagnetic waves and the distributed nature of the system were considered during the development of a two-hop routing protocol, which was implemented in the prototype’s software.

**Results:**

The outcomes of this research include a novel system architecture and routing protocol and a functional prototype based on commercial, off-the-shelf components. A proof-of-concept study was performed on a hemiplegic subject with paresis of the right arm. The subject was tasked with generating a fully functional palmar grasp (closing of the fingers). One node was used to provide this movement, while a second node controlled the activation of extensor muscles to eliminate undesired wrist flexion. The system was tested with the open- and closed-loop control algorithms.

**Conclusions:**

The system fulfilled technical and application requirements. The novel communication protocol enabled reliable real-time use of the system in both closed- and open-loop forms. The testing on a patient showed that the multi-node system could operate effectively to generate functional movement.

## Background

Functional electrical stimulation (FES) generates contraction of paralyzed or paretic muscles by activating a muscle’s neural supply [1]. Today, FES with surface electrodes is used to correct foot drop [2] and provide assistance for upper extremities [3]. Implantable FES systems, such as Actigait® [4] and Freehand® [5], are available on the market for the restoration of movement. However, FES is rarely used in clinical and home environments because of the complexity of its application, especially when several muscle groups must be activated to restore a complex function (e.g., walking). Electrical stimulation, however, was introduced more than 50 years ago [6], is widely and regularly used for therapy [7], and its cost/benefit has been recognized by clinicians and health care providers.

In this paper we present the architecture of a new wireless distributed FES system that can be used to develop and evaluate control strategies for both therapeutic and functional electrical stimulation applications. While the sensor and stimulation functionalities are entrusted to miniature wireless devices mounted on the body of the subject, the control algorithm runs on a powerful host computer.

The design of the new modular system follows clinical evaluation of multichannel stimulation systems that have contributed to functional recovery. Functional electrical therapy (FET) applied in acute and chronic stroke patients [8,9] demonstrated that effective long-term therapy with a practical FES apparatus can lead to the training of cortical structures [10]. FET applied for walking suggested similar improvements in functioning [11,12]. However, the hardware used in these applications was not practical if several stimulation channels or complex control were required. Our FES system was designed to be wearable, lightweight, easy to install, simple to maintain and flexible in operation. Essential components of our system support timely and selective activation of sensory-motor systems and feedback sensors.

The design of our modular system placed on the surface of the body follows the ideas of Loeb et al., who introduced the implantable stimulation system BION® [13]. The first version of the BION system provided distributed stimulation only and was powered and controlled using an external transmission coil in the vicinity of the implants. The second version of the system included sensing functionality through bidirectional communication [14]. Finally, they added a local rechargeable power source and processing to the implants, thus enabling extended daily use of the system without a bulky external transmission coil [15].

The concept of the technology described above inspired other researchers who tried to solve the problems of efficient communication and energy transmission [16-19] and integration of sensing functionality [20]. BION and similar technologies that use implants are advantageous for orthotic systems. The therapeutic use and testing of the possibilities for stimulation before actual placing of the implants relies on the transcutaneous systems, i.e., systems with surface electrodes. A suitable wire-based distributed FES architecture with surface electrodes was described by Andreu et al. [21].

Developments in the area of wireless sensor networks led to many new applications concentrated on monitoring human locomotion, sport results and therapy assessment [22,23]. Low-power, low-cost miniature sensing platforms have been used to remotely monitor individuals’ daily activities [24,25], post-operative care [26,27], and vital functions [28]. This research has led to a growing trend of extending from sensing to actuating functions using the same wireless medium. New applications employ wireless protocols to satisfy various demands such as low power, high data, and low latency throughput [29,30]. In the field of wireless functional electrical stimulation, several architectures have been presented [31-33].

We hypothesized that wireless communication could be used for reliable and timed transportation of sensor and stimulation data and enable stable closed-loop control of an FES system. Using commercial, off-the-shelf components, we designed the system prototype. We analyzed the influence of the signal attenuation, due to the signal’s propagation through the human body and in free space, on the wireless communication. Considering the distributed nature of the architecture, we proposed a new, simple and efficient wireless routing protocol. The proof-of-concept study was performed on a patient with hemiplegia. The results showed that the system could be used in both closed- and open-loop configurations.

## Methods

### Architecture of the system

**Figure 1:**
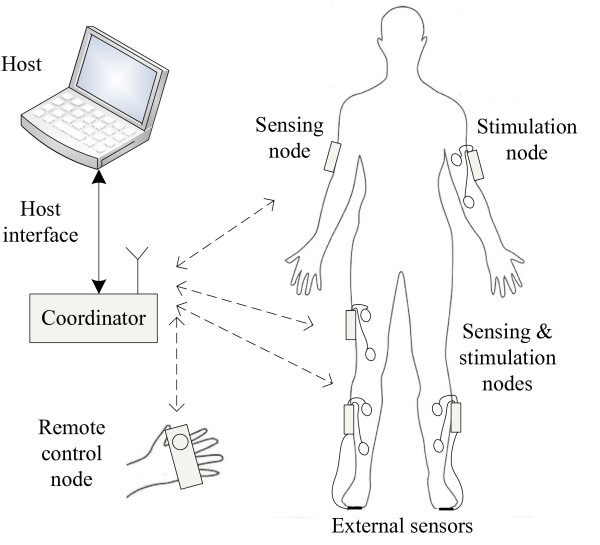
Architecture of the system, which consists of a set of devices with sensor/stimulation functionality mounted on the subject, remote control nodes, a coordinator node and a host that runs the control and assessment algorithms.

The general architecture of the system is presented in Figure 1. The system consists of a set of battery-powered peripheral nodes, one host computer, and one coordinator node. The peripheral nodes contain sensors and/or stimulators, depending on the desired application. The stimulation control algorithm runs on the host computer in open- or closed-loop form with sensor data inputs and calculated stimulation waveform outputs. The peripheral nodes exchange data with the host through the coordinator node. The communication is wireless between the peripheral nodes and the coordinator and wired between the host and the coordinator.

### Hardware prototype

The proposed architecture was successfully translated into a prototype system. The coordinator and the peripheral node designs are based on the Texas Instruments CC2430 microcontroller. The CC2430 is a true System on Chip (SoC) architecture that integrates in the same package an 8051 microcontroller core, a relatively large amount of RAM memory (8 K), and a Radio Frequency (RF) front-end. All registers in the RF front-end are accessible on the local bus of the microcontroller, which enables fast data exchange between the microcontroller core and the RF front-end.

The coordinator node includes the CC2430 microcontroller, RF amplifier and USB/serial transceiver. Because the size of the coordinator is not a limiting factor, an external high-gain antenna is used to enable a high-quality link and long-distance operation. Because the maximum current consumption is lower than 250 mA, the coordinator node is powered using the USB interface.

**Figure 2:**
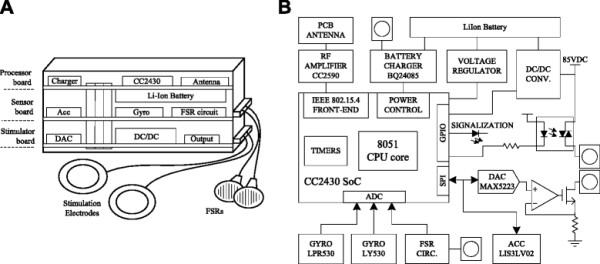
Hardware prototype of the peripheral node in full configuration: (A) the physical layout and (B) the electrical block diagram.

A peripheral node consists of two or three printed-circuit boards, depending on whether the node performs sensor and/or stimulation functions, packed in a sandwich structure (Figure 2A). The top board incorporates the microcontroller, battery management circuitry, and RF front-end. The middle board incorporates inertial Microelectromechanical Systems (MEMS) sensors and external sensor interfaces, while the bottom board serves as the stimulator. Each peripheral node is powered using a Li-ion 500 mAh battery mounted between the top and middle boards. In the full configuration (all three boards), the peripheral node’s dimensions are 70x25x30 mm and its mass is 45 g. If the node is operated as a sensor or stimulator, but not both, the dimensions are slightly smaller.

The electrical block diagram of the peripheral node in full configuration, with both sensing and stimulating functions, is presented in Figure 2B. The microcontroller integrates an 8-channel 12-bit AD converter, SPI controller, and several output compare units for PWM generation. The RF amplifier is connected to a small inverted-F PCB antenna. The sensor board contains a digital 3D accelerometer LIS3LV02 (ST) and a combination of two pitch-roll LPR530 (ST) and yaw rate LY530 (ST) analogue gyroscopes that form a miniature inertial measurement unit with six degrees of freedom. The sensor board also includes circuitry for conditioning signals from resistive sensors, such as FSRs and flex-force goniometers. The stimulation board has a DC/DC step-up converter that produces 85 V DC. The electrodes are driven by two current-controlled output channels (only one channel is presented in Figure 2B) with optotriac-enabled negative pulse compensation. The current amplitude is determined by a 10-bit SPI DAC MAX5223 (Maxim) and ranges from 0–70 mA. The duration of the stimulation pulses ranges from 10–1000 μs with 8-bit resolution. The frequency and duty ratio of the stimulation pulses are controlled by the output compare units of the microcontroller timers. The efficiency of the stimulator is approximately 40%, and the maximum average output power is 0.7 W.

### Wireless communication

The wireless communication of the system is based on the IEEE 802.15.4 physical standard. The usability and reliability of the system rely on the system’s ability to achieve periodic, delay-free communication between every peripheral node and the coordinator.

**Figure 3:**
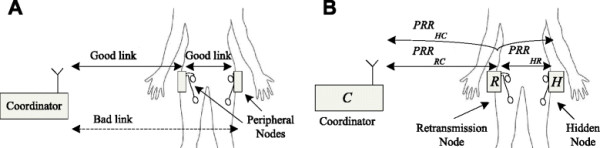
Propagation of signals through the human body: (A) link quality differences, and (B) packet reception rate calculation.

If interference with other systems is neglected, the main reason for a break in communication between the coordinator and a peripheral node is high signal attenuation through the human body. The high attenuation factor of human tissue is the result of the energy absorption of water, which is present in the 2.4 GHz band [34], and is significantly higher than the attenuation factor of air. Yet the impact of the attenuation depends on the relative positions of the node, coordinator, and human body. If the distance between the subject and the coordinator is large, a node hidden behind the body may be unreachable by the coordinator, while a visible node can reach both the coordinator and the hidden node (Figure 3A). Considering this scenario, the communication between the coordinator and the hidden node can be established indirectly through a retransmission route involving a node that is visible to the coordinator and the hidden node (Figure 3B).

The statistical measure of the communication link quality between nodes X and Y is called the packet reception rate and is denoted as *PRR*_*XY*_. *PRR*_*XY*_ is defined as the probability of correct reception of packet data sent from node X to node Y. If we assume that the channels are statistically independent and the links are symmetric, the packet reception rate for the whole retransmission route from the hidden node to the coordinator can be calculated as

(1) $$PRR_{\mathrm{HC}}={PRR}_{\mathrm{HR}}\times {PRR}_{\mathrm{RC}}$$

where *PRR*_*HR*_ accounts for the probability of a correct packet transmission from the hidden node to the retransmitting node, and *PRR*_*RC*_ accounts for the probability of a correct packet transmission from the retransmitting node to the coordinator node [35]. Considering the physical properties of the system, there is a reasonable expectation that the link qualities of peripheral nodes mounted on the subject are time-invariant and exhibit a stable *PRR*. Additionally, when the transmission power of the nodes is adequately high, which is assumed for this system, the packet reception rate among the peripheral nodes can be considered to be close to one. This assumption leads to the following simplification of equation (1)

(2) $$PRR_{\mathrm{HC}}={PRR}_{\mathrm{RC}}$$

which suggests that the equivalent reception rate for a two-hop link depends mainly on the sub-link between the coordinator and the retransmission node.

### Routing - retransmission request routing protocol (R^3^P)

**Figure 4:**
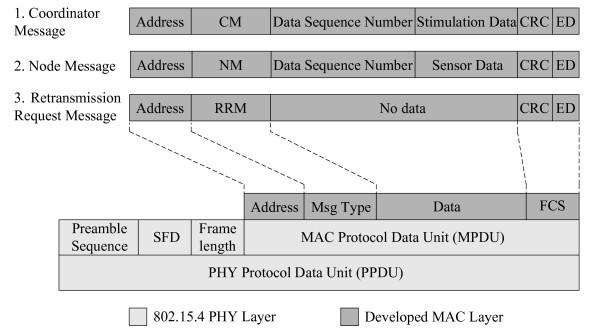
Frame format including three basic types of messages.

In the ideal case with no link breaks, two messages are transferred: the coordinator message and the node message (Figure 4). The coordinator message is sent from the coordinator to the addressed peripheral node. In the general case when a node integrates stimulation and sensor functions, the coordinator message contains stimulation data and the data sequence number for the requested sensor data. After receiving the coordinator message, the peripheral node replies to the coordinator with a message containing the requested sensor data and data sequence number. The node message serves as an acknowledgement for the coordinator and is sent even if the peripheral node does not contain sensor functions. After receiving the acknowledgement, the coordinator establishes the connection with the next peripheral node according to the round-robin scheduling principle. In addition to the functional data, each message ends with a frame check sequence (*FCS*) field that contains a cyclical rule check (*CRC*) flag and energy detection (*ED*) data, which is an estimate of the received signal power within the IEEE 802.15.4 channel bandwidth [36]. The *FCS* field is not part of the transferred data; the field is generated locally based on the RF receiver logic after receipt of the message.

As illustrated in Figure 3B, in the case of a link breakage, two-hop routing can be used to achieve communication with a distant node. The main demand for the routing algorithm is that all nodes in the network receive all communication messages no matter if they are addressed or not. The last correctly received message is stored in peripheral node’s local buffer. The routing protocol is implemented when the coordinator issues an additional message, called the retransmission request message (RRM) to the selected retransmission node, as shown in Figure 4. After receiving the RRM, the selected node resends the content of the last received message in its buffer without any additional processing.

**Figure 5:**
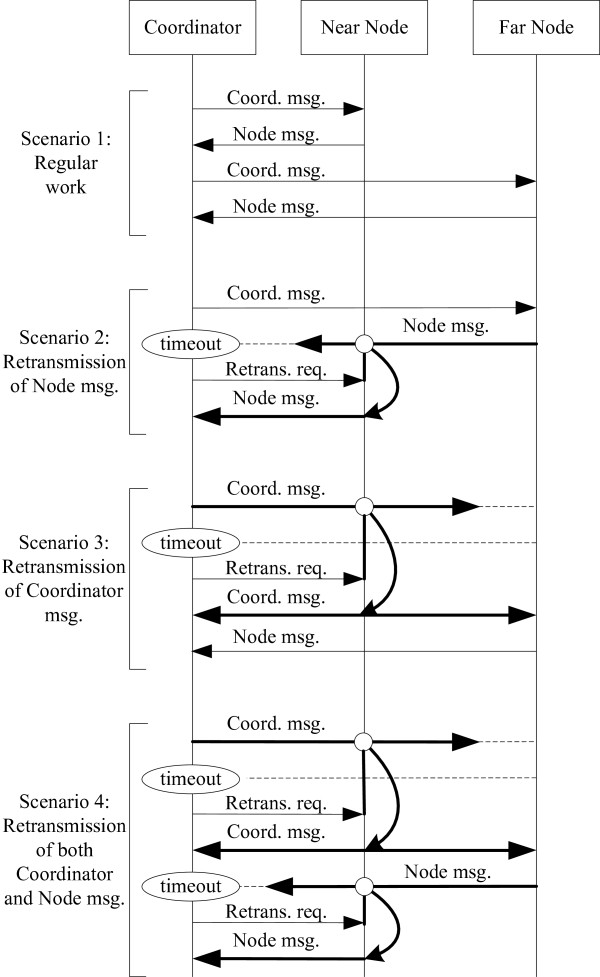
Network communication diagram with four different scenarios that cover situations encountered during regular network function.

Although a network usually comprises several nodes and a coordinator, the routing algorithm is further analyzed in a simplified case of one coordinator and two nodes, without the loss of generality. The basic assumption is that one of the nodes, called the near node, has ideal, high-quality links with both the coordinator and the other node. The second node, called the far node, is assumed to have a realistic link with the coordinator, which can be broken. Four possible scenarios of the communication between the coordinator and the far node are presented in Figure 5.

Scenario 1 represents regular operation, when both the near node and the far node have high-quality links and visibility with the coordinator. The coordinator periodically requests and receives data from both nodes at predefined time intervals.

Scenario 2 represents a link break between the far node and the coordinator. In this case, the far node receives a message from the coordinator and replays with its data message. Because of the high-quality link between the near and far nodes, the near node receives the message from the far node and stores the message in its message buffer, but the coordinator does not receive the far node’s message. After a predefined amount of time, the coordinator realizes that the communication has failed and issues a retransmission request message to the near node, which is assumed to have a high-quality link with the far node. After receiving the retransmission request message, the near node transmits the buffered message, which is the same message the far node tried to send to the coordinator in the previous transmission. Because of the high-quality link between the near node and the coordinator, the node message is successfully delivered.

Scenario 3 covers a break in communication during the sending of the coordinator message. Because of the high-quality link between the near node and the coordinator, the near node receives and buffers the coordinator message, but the far node does not receive the message. After a predefined amount of time, the coordinator issues a retransmission request message, which induces the near node to resend the message issued by the coordinator in the last transmission. Because of their high-quality links with the near node, both the coordinator and the far node receive the retransmitted coordinator message. Then, the far node sends its node message to the coordinator, just as in scenario 1. After the coordinator receives its own retransmitted message, the coordinator waits for a message from the far node, just as in scenario 2.

Scenario 4 considers a break in transmission of the message from the far node to the coordinator. If the coordinator receives the retransmitted coordinator message but does not receive a message from the far node after a predefined amount of time, the coordinator issues a second retransmission request message to the near node, which will resend the message from its buffer to the far node.

According to equation (2), selection of the retransmission node can be implemented with a simple rule: The peripheral node that has the highest *PRR* with the coordinator should be chosen as the retransmission node. Inspired by the research in [37], *PRR* can be represented as

(3) $$PRR=f\left(ED\right)\text{,}$$

where *f* is the monotonous rising function and the *ED* is received with each new message. This result means that the higher the *ED*, the higher the *PRR*. The task of the coordinator is to continuously monitor the *ED* levels of all links to determine the retransmission node when needed.

The proposed routing protocol is effective when a near node exists that can act as a good mediator between the coordinator and the other (far) nodes. Because the system usually consists of several nodes distributed around the subject’s body, this requirement is typically fulfilled. However, if the requirement is not fulfilled, a dummy node can be added and serve as an appropriate retransmission node.

### Software

The software of the system is divided into three logical and functional entities: coordinator firmware, peripheral node firmware, and host computer control software.

The coordinator firmware implements the routing algorithm and the efficient transport of the messages between the host computer and the peripheral nodes. Message correctly received from the node is directly transferred to the host, and the data sequence number is extracted from the message and saved into the coordinator’s memory. The data sequence number determines the last received packet of data in the time sequence. Before issuing the new message, the coordinator increases the data sequence number to request new data. In the case of incorrect node message reception, the data sequence number is not increased.

The peripheral node firmware handles the acquisition of the sensor data, the RF communication with the coordinator, and the stimulation pattern. Because of possible breaks in the transmission, there is a need for local buffering of the sensor data.

The host receives node messages containing sensor data in the order the messages are acquired by the coordinator. The sensor data are saved in circular FIFO buffers reserved for all signals of all nodes. In the real environment, the routing algorithm minimizes data losses, but delays can occur. Control strategies in functional electrical stimulation are based on various processing techniques, such as fuzzy logic, rule-based control, and neural networks. Many of these methods process time-series data. To hide the delays from the control algorithm, a prediction phase is placed after the data acquisition phase. One pass through the control algorithm provides stimulation data for the next time period. Calculated stimulation data are sent to the coordinator, which passes them to the peripheral nodes.

In addition to the control algorithm, the host computer control software contains a graphical user interface. Additional modules, such as database logging and a communication interface for telemedicine applications, can be added.

## Results

### Experimental testing

**Figure 6:**
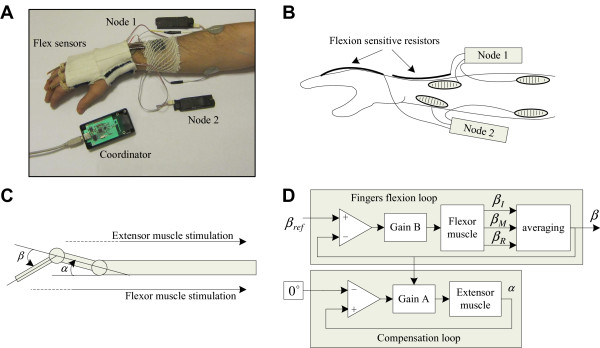
Experimental setup: (A) photo of the system mounted on the subject, (B) schematic drawing of the setup, (C) functional model of the arm, and (D) control algorithm model.

The proof-of-concept study was performed on a hemiplegic subject with paresis of the right arm. The subject signed the informed consent approved by the local ethics committee. The subject’s right hand was in a resting position with fingers slightly flexed (approximately 20 degrees) and could not be voluntarily opened or closed. The patient was moderately spastic. The subject’s task was to generate finger movement appropriate for a functional palmar grasp. The term “functional” referred to having fingers flexed around an object but an unflexed wrist. This task was selected because the task required sensing finger and wrist joint angles and concurrent stimulation of muscles, which resulted in finger flexion and control of the wrist joint. Namely, the stimulation of finger flexors, which are located in the forearm, also resulted in wrist flexion. Wrist flexion causes tenodesis, which contributes to finger extension and compromises the functional grasp. To prevent unwanted effects, the wrist extensor muscles (on the volar side of the forearm) were stimulated concurrently with the deep and superficial flexor muscles (on the dorsal side of the forearm). Figure 6A shows the experimental setup, and Figure 6B shows the model of the system. The system comprised two nodes: one for the wrist extension and one for the finger flexion. Each node featured sensing and stimulation. Node 1 was used for the stimulation of the wrist extension, which moved the hand up and kept the angle *α* within predefined boundaries, and Node 2 activated the flexor muscles, which flexed the fingers up to a predefined angle of *β* (Figure 6C). Node 2 was connected to three flex-force sensors mounted on the index, middle and ring fingers. Each sensor’s change in resistance was strongly correlated with the angle. Node 1 was connected to one flex sensor mounted on the upper side of the wrist. The control algorithm was presented by two closed loops (Figure 6D).

The main loop implemented proportional control of the finger flexion stimulation. The input to the controller was the reference angle *β*_*ref*_, and the output was the effective flexion angle *β*, calculated as the averaged value of angles from the index finger (*β*_*I*_), middle finger (*β*_*M*_) and ring finger (*β*_*R*_). The second loop compensated for hand bending and ensured that the wrist angle *α* remained zero during the finger flexion. Measurements showed that the most convenient way to compensate for hand bending was to modulate the loop gain with the actual angle *β*. In other words, a larger degree of finger flexion required stronger compensation of the wrist extension stimulation.

**Figure 7:**
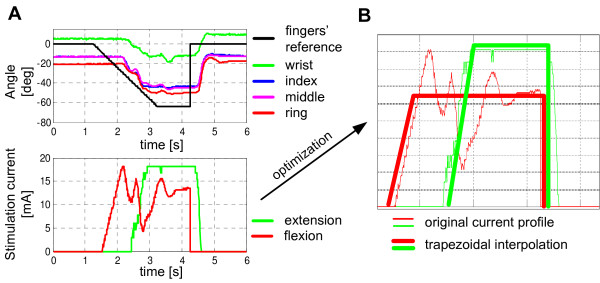
Results of the proportional control algorithm: (A) time diagrams of the referent angle, wrist and finger angles and stimulation current waveforms resulting from one trial and (B) trapezoidal approximation of the resulting current waveforms.

The result of the applied feedback control algorithm is presented in Figure 7A. The system was excited with a trapezoidal reference angle waveform. Because the neuromuscular system possessed a transport delay, using a pure proportional regulator caused expected oscillations. Nevertheless, the output showed a tendency toward attenuation of oscillations. Measured current waveforms were then optimized to be used as reference patterns for the open-loop control. The optimization was implemented as an automatic procedure in the software running on the host computer. The two basic constraints of the optimization were (a) the total charge amount must be unchanged, and (b) the resulting waveform should be of a trapezoidal shape (Figure 7B).

**Figure 8:**
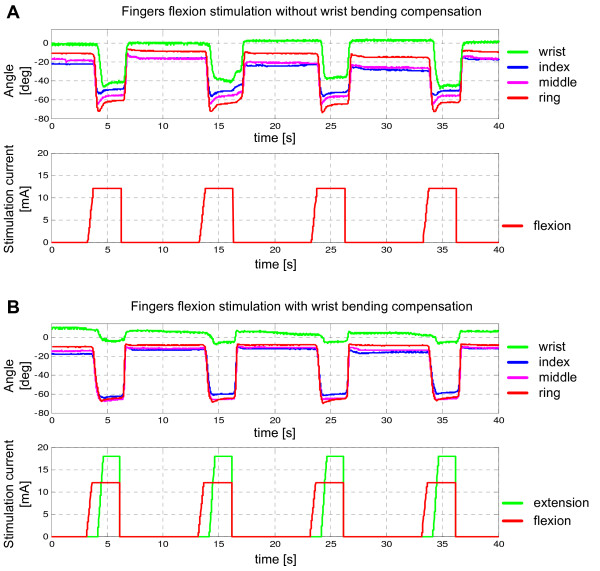
**Joint angles and current pulse amplitude profiles for open-loop control: (A) stimulation of the flexor side of the forearm only (no wrist compensation), and (B) stimulation of finger flexors and wrist compensation. In both cases, the amplitude profiles used were from the initial testing with the feedback (Figure.**7**)**

The trapezoidal stimulation current waveforms determined automatically using the results from the closed-loop control were then used for the open-loop stimulation. Figure 8A shows the finger and wrist angles when only the flexor muscles were stimulated. As expected, a significant flexion of the wrist accompanied the flexion of the fingers. Figure 8B shows the same set of angles when the compensation of the extensor muscles was used. For this test, the dorsal and volar sides of the forearm were stimulated with the distributed 2-node system.

## Conclusion

We presented the architecture for a complex multi-channel wireless distributed system and developed a prototype that can be used to develop and evaluate functional electrical stimulation algorithms. The system uses a central unit to implement a hierarchical control of multiple self-powered peripheral nodes. Each node comprises up to two stimulation outputs, inertial sensors, and an interface for force-sensing resistors. The inter-node communication and communication with the central unit have been designed upon the physical layer of the IEEE 802.15.4 standard for low-power, ad-hoc wireless sensor networks. Physical properties of signal propagation through the human body together with spatial characteristics of the system setup were considered during the development of the wireless communication, and a novel Retransmission Request Routing Protocol (R^3^P) is presented.

The protocol is based on the master–slave hierarchy where every communication is initiated and controlled by the coordinator. Two-hop routing is implemented by issuing a short retransmission request command to the selected retransmission node. From the node side, the computational efficiency and speed of the protocol lies in the fact that the retransmission node does not make any calculations and only resends the last received message. From the coordinator side, the protocol is implemented through the state machine with four naturally concatenated scenarios. To select the retransmission node, the energy detection levels of all possible candidates are compared. Because the energy detection levels are generated and measured locally, the throughput of the system is not decreased by the protocol. The main demands on the protocol are that each node in the network receives every communication message, even if the message does not address that node specifically, and that the nodes are well-distributed physically around the subject’s body. The first demand is fulfilled by the software implementation, and the second can be easily fulfilled by adding an appropriate dummy node that will serve as a good retransmission node.

The system was tested in a situation that required the use of sensors and at least two stimulation units to provide better clinical operation. The tests conducted using a single unit showed clear interference (i.e., undesirable movements during stimulation that compromised the therapeutic effects of stimulation). The tests with compensatory stimulation and feedback emphasized the difficulty of applying closed-loop control; namely, the unavoidable delays in the muscle responses led to an overshoot of stimulation strength and undesirable movement responses. Therefore, we developed an automatic procedure that used the data from the initial short series of closed-loop tests to generate a stimulation sequence that could minimize the overshoot and contribute to the effective operation of the stimulation system.

All three phases of the experiment (closed-loop test, optimization, and open-loop test) were conducted sequentially without delay and used the same setup and electrode placement. This outcome was possible because all control, optimization, and data logging algorithms were running in real-time on a powerful host computer, and the results were visible online thorough a graphical user interface. We think that the main advantages of the proposed system compared with other systems used in FES therapy and research are the integration of a fixed computer’s processing power, the convenience of miniature wireless sensors and actuators, and the intuitiveness of a graphical user interface. This system can contribute to the faster development of new FES control strategies. Our future work will focus on using this system to develop closed-loop control strategies that rely on complex data processing techniques.

## Competing interests

The authors declare that they have no competing interests.

## Authors’ contributions

NSJ was involved in designing the system’s hardware and software, developing the prototype, recruiting the human subject, conducting the experiment, collecting and analyzing data, interpreting the results, and writing the manuscript. LVS was involved in designing the system’s hardware and software and provided critical revisions of the manuscript. DBP was involved in defining the system requirements, recruiting the subject, interpreting the results, and revising the manuscript. All authors have read and approved the final manuscript.
